# N6-Methyladenosine Related Long Non-Coding RNAs and Immune Cell Infiltration in the Tumor Microenvironment of Gastric Cancer

**DOI:** 10.1186/s12575-021-00152-w

**Published:** 2021-08-01

**Authors:** Zhong lin Yu, Zheng ming Zhu

**Affiliations:** 1 Department of Gastrointestinal Surgery, The Second Affiliated Hospital of Nanchang University, 1 Minde Road, Nanchang, 330006 Jiangxi China; 2 Key Laboratory of Molecular Medicine of Jiangxi Province, Nanchang, 330006 Jiangxi China

**Keywords:** Bioinformatics analysis, N6-methyladenosine, lncRNAs, Immune cell infiltration, Gastric cancer

## Abstract

**Aim:**

To illustrate the influence of N6-methyladenosine long non-coding RNAs and immune cell infiltration in gastric cancer.

**Methods:**

We downloaded workflow-type data and clinical data from The Cancer Genome Atlas project. The relationship of lncRNA and m6A was identified. Kyoto Encyclopedia of Genes and Genomes gene expression enrichment analysis was performed. Lasso regression was utilized to construct a prognostic model. Survival analysis to explore the relationship between m6A lncRNA and clinical survival data. Differential analysis of the tumor microenvironment and immune correlation analysis to determine immune cell infiltration levels and their correlation with clinical prognosis.

**Results:**

Co-expression analysis indicated that lncRNA expression was associated closely with m6A. m6A-lncRNAs were partially highly expressed in tumor tissue and could be used in a prognostic model to predict GC prognosis, independent of other clinical characteristics. “ADIPPOCYTOKINE SIGNALING PATHWAY” was most significantly enriched according to GSEA. ACBD3-AS1 was overexpressed in tumor tissue. Naïve B cell, Plasma cells, resting CD4 memory T cell were highly infiltrated tissues in cluster 2, while Macrophages M2, resting Mast cells, Monocytes, regulates T cells were lowly in cluster 1. All related scores were higher in cluster 2, indicating a lower purity of tumor cells and higher density of immune-related cells in the tumor microenvironment.

**Conclusion:**

m6A lncRNA is closely related to the occurrence and progression of GC. The corresponding prognostic model can be utilized to evaluate the prognosis of GC. m6A lncRNA and related immune cell infiltration in the tumor microenvironment can provide novel therapeutic targets for further research.

## Introduction

According to global cancer statistics, there were 1,089,103(5.6%) new cases and 768,793(7.7%) deaths from gastric cancer in 2020. The incidence and mortality rate of gastric cancer ranked fifth and forth, respectively [1]. With a multidisciplinary team approach and the application of postoperative hyperthermic intraperitoneal chemotherapy, the five-year survival rate of gastric cancer can be optimized to a certain extent. However, effective treatments for advanced metastatic GC need to be explored. One of the fundamental factors that accelerates the occurrence and progression of GC is the accumulation of epigenetic transformations [2].

m6A is related to several mRNA metabolism pathways involved in mRNA translation and decay; together with 5-methylcytosine and pseudouridine, m6A constitutes the epitranscriptome and affects protein synthesis [3]. Aberrant m6A modification stimulates tumor stem cell self-renewal, which intricately contributes to the progression of tumorigenesis [4]. There is increasing evidence that long non-coding RNAs (lncRNAs) are involved in a multitude of cancers based on their large number and expression specificity, and facilitating the elucidation of functional cancer-associated transcripts would be beneficial for the development of therapeutic interventions against cancer [5, 6].

m6A-methylated lncRNA has important regulatory effects on several biological and pathological processes. m6A mediates immunotherapy and prolongs survival by modulating gene expression and splicing, which causes changes in lactate levels in the tumor microenvironment [7]. Furthermore, knockdown of METTL14 heightened proliferative and invasive potential of GC cells and promoted tumorigenicity and metastasis via the PI3K/AKT/mTOR signaling pathway [8]. However, the aberrant expression and m6A methylation of lncRNA in GC remain unknown. Therefore, the formation of a transcriptome map of the expression and m6A modification profiles of lncRNA in GC is of great significance to gain understanding of the mechanisms by which lncRNA affects the prognosis of GC.

Immune cell score analysis improve the prognostic evaluation of gastrointestinal cancer patients. Immune-risk score provides prognostic evaluation and adjuvant treatment guidance, which enhances our comprehension of the relationship between chemotherapy and the immune system to broaden the claim of immunotherapy [9]. Immune scoring system improves prognosis prediction accuracy of TNM stage and distinguish GC patients with better prognosis, which might help us identify GC patients who would get help from adjuvant chemotherapy [10]. The immunoscore is a measure of cytotoxic immune reactions, which guide the formulation of treatment strategies, and can improve the prognosis of collision tumors [11]. Regrettably, few researchers have investigated the correlation between m6A lncRNA and immune cell infiltration in GC. Thus, it is important to analyze immune cell infiltration in the tumor microenvironment and clarify its relationship with the clinicopathological parameters of gastric cancer.

## Materials and Methods

### Sample Data Acquisition and Collation

We downloaded gastric cancer gene expression profiles and clinical data from the TCGA through the Genomic Data Commons Data Portal [12] (https://portal.gdc.cancer.gov/) up to March 2021, the public database included the expression profiles of 375 gastric cancer tissues and 32 normal tissues derived by HIseq-FPKM. Then, we built the mRNA matrix via PERL software (https://www.perl.org/) and used the corresponding script to organize transcriptomic data and transform gene IDs. Finally, the same software and specific script were utilized to process the clinical data. When we installed the software, the language of the installation path was set as English, and there were no spaces included. Based on the guidelines released by the National Cancer Institute in December 2015 (https://cancergenome.nih.gov/publications/publication guidelines), our research did not require the approval of an ethics committee.

### Identification of m6A Related lncRNAs

To distinguish mRNAs and lncRNAs by utilizing the collated transcriptomic data, we constructed a gene expression matrix and human configuration file containing the expression levels of m6A-related lncRNA gene profiles using PERL software. By running the corresponding script file, we obtained the attributes of related genes and obtained the respective mRNA and lncRNA gene expression data. Then, the gene IDs were transformed to gene names based on information in the Ensembl database (http://asia.ensembl.org/info/data/index.html). According to the m6A-related gene type (“writers”, “readers”, “erasers”) and the corresponding gene name, m6A-related gene expression data were extracted with the limma package in R software (https://www.r-project.org/). Next, co-expression analysis was carried out to identify the correlation among m6A-related gene expression and lncRNAs. According to the correlation coefficient, we obtained regulatory hypotaxis between the two types. By this method, we acquired the expression data of m6A-related lncRNAs through the limma package in R software (http://bioconductor.riken.jp/packages/3.0/bioc/html/limma.html). Moreover, a network plot was depicted via the igraph package to visualize the correlation. Expression data of m6A-related lncRNAs were combined with clinical survival data via the limma package. Prognostic-related lncRNAs were extracted, and the confidence interval and hazard ratio were calculated by the survival package. Visualization of univariate Cox regression analysis was achieved by drawing forest plots. The differential expression data of prognosis-related m6A lncRNAs between tumor and normal tissues were acquired through the limma package, pheatmap package, reshape2 package and ggpubr package in R software. Differences with P < 0.05 were considered statistically significant. To visualize the differences in expression, we drafted heatmaps and boxplots.

### The Role of m6A lncRNAs

First, we sorted prognosis-related m6A lncRNAs into two subtypes, cluster 1 and cluster 2, via ConsensusClusterPlus and limma packages according to the expression of lncRNAs (https://bioconductor.org/install/). The calculation method was clusterAlg = “km” and clusterNum = “2”. We executed survival analysis according to lncRNA subtypes via survminer and survival packages to evaluate the prognostic value of m6A lncRNAs. To identify differentially expressed prognosis-related lncRNAs in each cluster and analyze the relationship between lncRNAs and clinicopathological parameters, the pheatmap package was utilized, and a heatmap was depicted. Differential expression of target genes in related subtypes and different types of tissue was identified via the limma package. The standard names of the target genes were obtained from NCBI (https://www.ncbi.nlm.nih.gov/). To clarify the correlation between the target genes and prognostic m6A lncRNAs in gastric cancer, gene correlation analysis was conducted via the limma package. The difference was considered statistically significant when *P* < 0.05.

### The Role of Immune Cell Infiltration and the Tumor Microenvironment

To explore and calculate the infiltration level of different immune cell types in the samples, the preprocessCore, limma and e1071 packages were utilized, and the relative number infiltrating immune cells was obtained. The analysis of the tumor microenvironment was carried out via the estimate and limma packages, and the stromal score, immune score and estimation core were determined. The above scores were inversely related to tumor purity. Differential analysis of immune cell infiltration in different clusters was carried out by the limma package, and vioplot was used to visualize the differences. Additionally, the infiltration level of each type of immune cell in different clusters of gastric cancer was analyzed via the limma package, and a boxplot was drawn. Immediately, we conducted differential analysis of the tumor microenvironment in different subtypes of samples by the limma package according to the immune score, estimate core and stromal score, and corresponding boxplots were generated to further explore the purity of tumor cells in different types. Then, gene set enrichment analysis (GSEA) (https://www.gsea-msigdb.org/gsea/index.jsp) was conducted to clarify the differences in related functions and pathways in different samples, and import data were obtained via PERL software. Related scores and plots were obtained to evaluate whether gene set enrichment was dynamic in different clusters (c2.cp.kegg.v.7.2.symbols.gmt, cluster.cls#C2 versus C1). Each sample was defined as either “H” or “L,” depending on whether it was a high-or low-risk cluster of prognosis-related lncRNAs. The number and type of permutations were set at “1000”, “no Collapse” and “phenotype”, respectively. The gene list ordering mode was “descending”. The gene list sorting mode was “real”. The metric for ranking genes was “Signal2Noise”. The normalization mode was “meandiv”. FDR < 0.05 indicated that the difference was statistically significant.

### m6a-related lncRNA Prognostic Model

Lasso regression was carried out to construct a prognostic model. All samples were divided into a high-risk group and a low-risk group according to the median value of the risk score of prognostic m6A lncRNAs. Training (50%) and test (50%) groups were used for the lasso regression, and related plots were obtained. The survival curves of different groups were depicted, in which high-risk and low-risk groups were compared. To evaluate the accuracy of our model for predicting the survival of patients with the disease, a corresponding ROC curve was obtained via the time ROC package. A risk curve was generated in line with the risk score, survival status and risk associated with m6A lncRNAs. Independent prognostic analysis was conducted to evaluate whether our model was independent of other clinical prognostic factors that affect patient outcomes. Multivariate and univariate analyses were carried out, and the hazard ratio was calculated. Model validation for clinical groups was utilized to test and verify whether our model could be applied to different clinical groups. Risk and clinical correlation analysis was carried out to clarify related high-risk and low-risk m6A lncRNAs and expound the correlation of clinical characteristics and our prognostic risk model. A heatmap was generated via the pheatmap package and limma package. Boxplots of risk and clinical relevance were obtained to evaluate the relationship between risk and the clinical data. Genetic differential analysis was performed to assess the expression difference of target genes in different risk groups in our model in gastric cancer. Correlation analysis of risk and immune cells was conducted to evaluate the relationship between immune cells and the risk score. A scatter plot was generated to visualize this nexus and assess whether immune cells were beneficial or detrimental.

## Results

### Identification of m6A Related lncRNAs

**Fig. 1:**
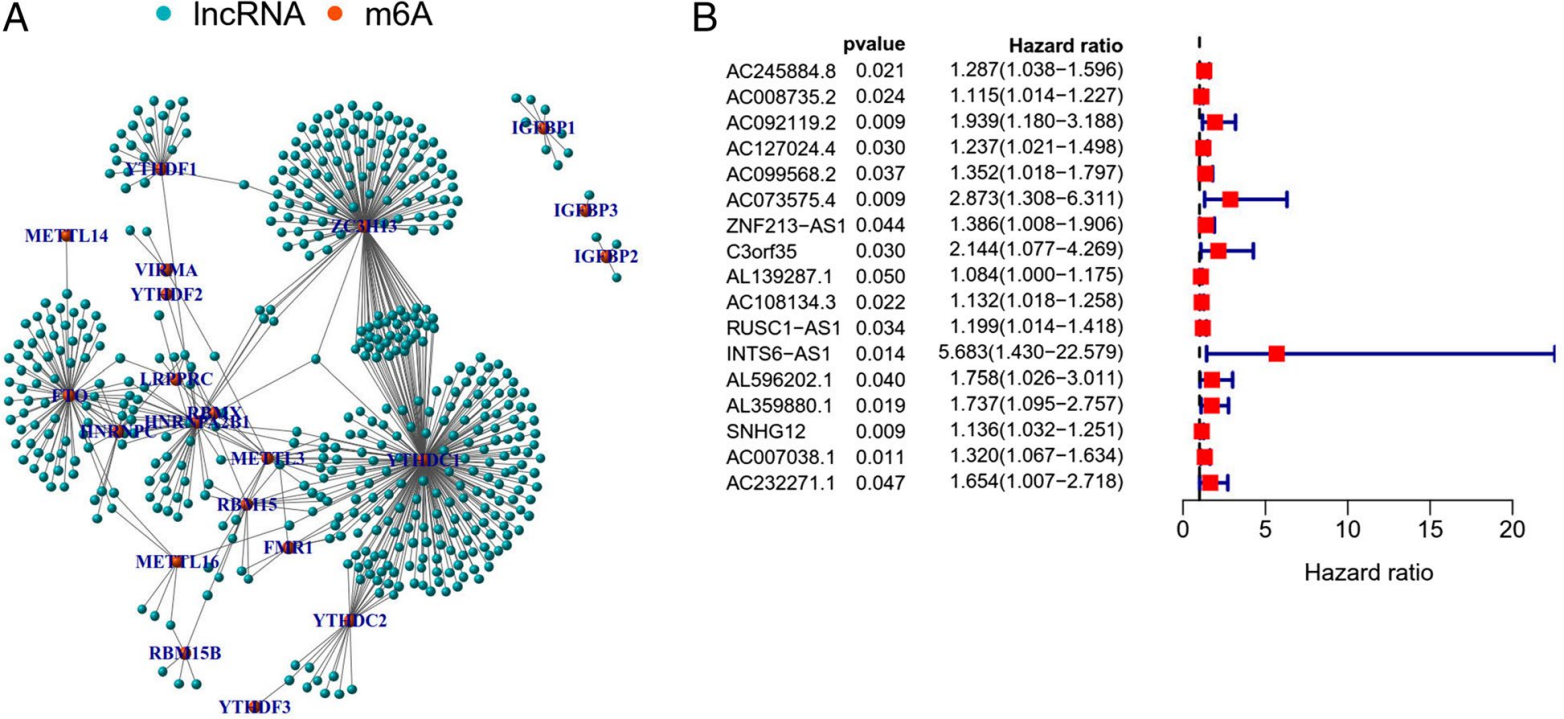
The expression of m6A-long noncoding RNAs (lncRNAs) and their role in the prognosis of GC patients. **A**: Network plot of correlation among m6A related gene expression and lncRNA. **B**: forest plot of univariate cox regression analysis. Prognostic related lncRNAs data was extracted and the confidence interval and hazard ratio were calculated. Red represents high risk, while green represents low risk

**Fig. 2:**
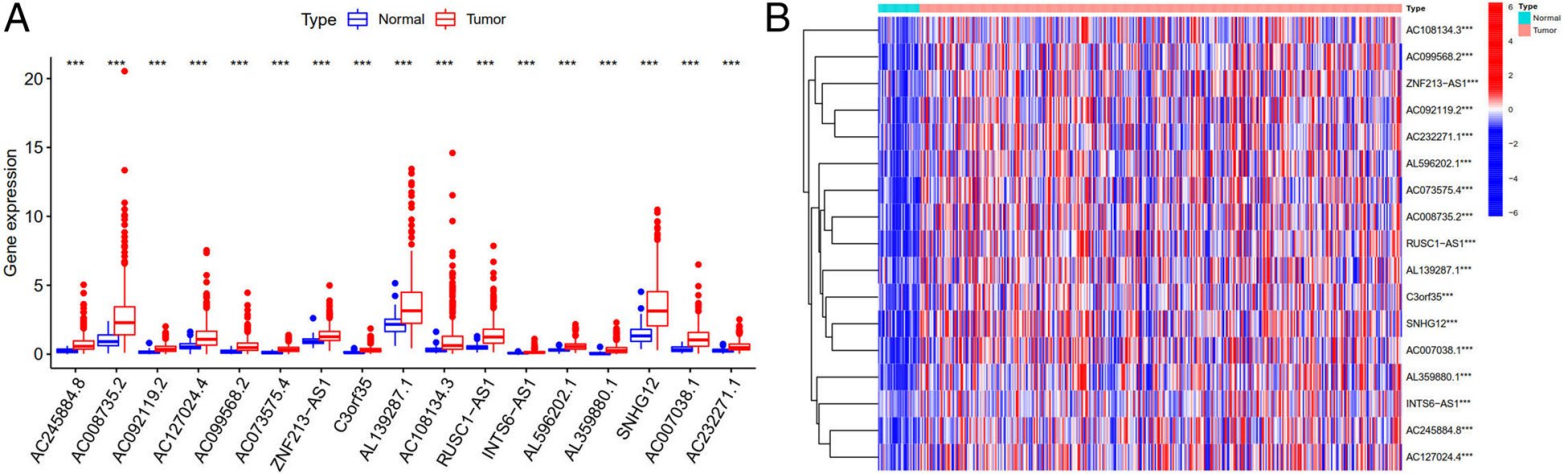
Difference expression of m6A prognostic related lncRNAs. **A**: Boxplot of the difference expression of m6A prognostic related lncRNAs among tumor and normal tissue. *:*P* < 0.05; **:*P* < 0.01; ***:*P* < 0.001. **B**: Heatmap of the difference expression of m6A prognostic related lncRNA among tumor and normal tissue. *:*P* < 0.05; **:P < 0.01; ***:P < 0.001. Red represents high expression, while blue represents low expression. The abscissa represents the sample, while the ordinate represents prognostic related lncRNA

m6A-related gene expression data were extracted from collated transcriptomic data to discriminate mRNAs and lncRNAs. Next, co-expression analysis was performed to identify the correlation between m6A-related gene expression and lncRNAs. A network plot was generated to visualize this correlation (Fig. 1A). The univariate Cox regression analysis was visualized as a forest plot (Fig. 1B). LncRNAs were considered to be m6A prognosis-related lncRNAs when P < 0.05. The differential expression of prognosis-related m6A lncRNAs between tumor and normal tissues was acquired, and heatmaps and box plots were obtained, as shown in Fig. 2A and B. There were 17 differentially expressed m6A prognosis-related lncRNAs between tumor and normal tissues. Some of these lncRNAs were highly expressed in tumors, while others were highly expressed in normal tissue (*P* < 0.05).

### The Role of m6A lncRNAs

**Fig. 3:**
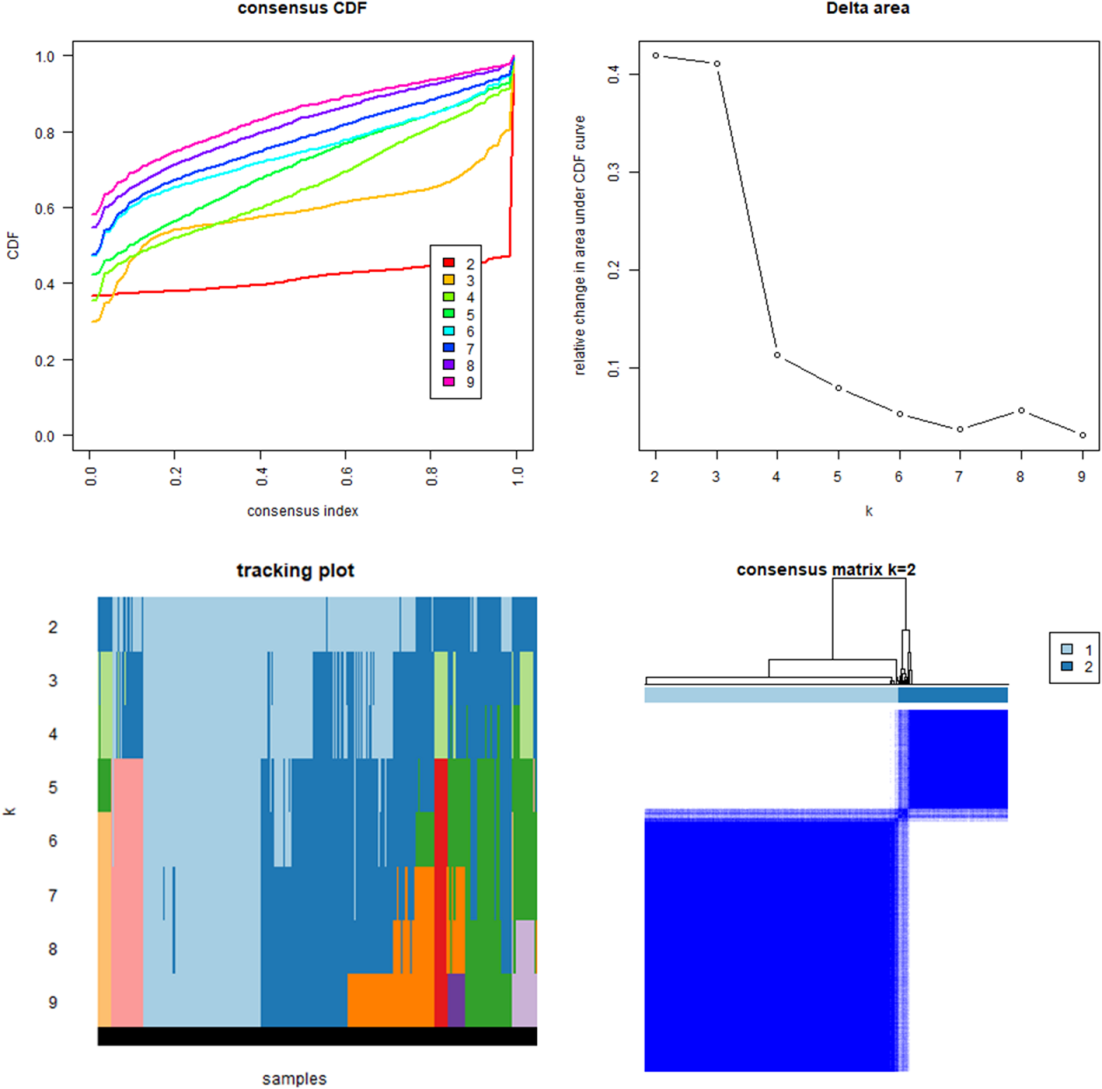
The type of prognostic related m6A lncRNAs. According to the expression of lncRNAs, when K = 2, there were least cross-mixing part between the two types and the CDF value was lowest, so we classified lncRNAs into two types: cluser 1 and cluster 2

**Fig. 4:**
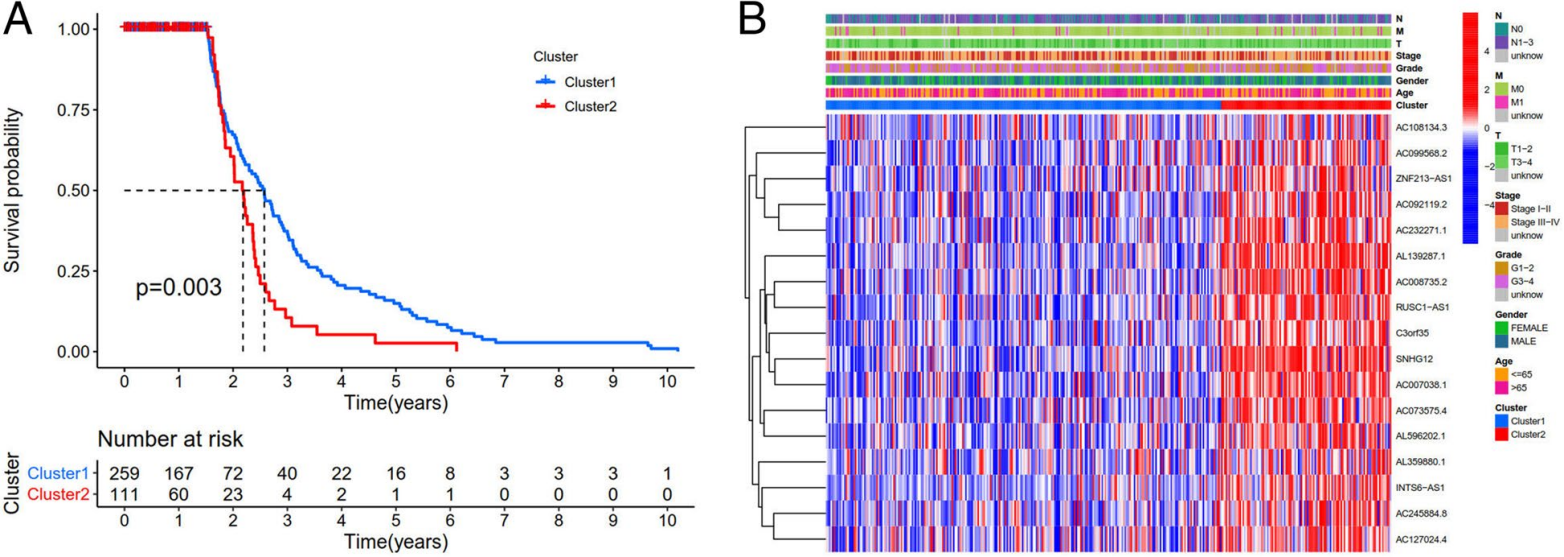
Survival analysis of prognostic related m6A lncRNAs and relationship with clinicopathological parameters. **A**: Survival analysis according to subtypes of lncRNAs, the 5 year survival rate of cluster 1 was higher, *P* = 0.003. **B**: Heatmap of difference expression of prognostic related lncRNAs and relationship with clinicopathological parameters in different cluster. *: *P* < 0.05. Red represents high expression, while blue represents low expression. The abscissa represents the sample, while the ordinate represents prognostic related lncRNA

**Fig. 5:**
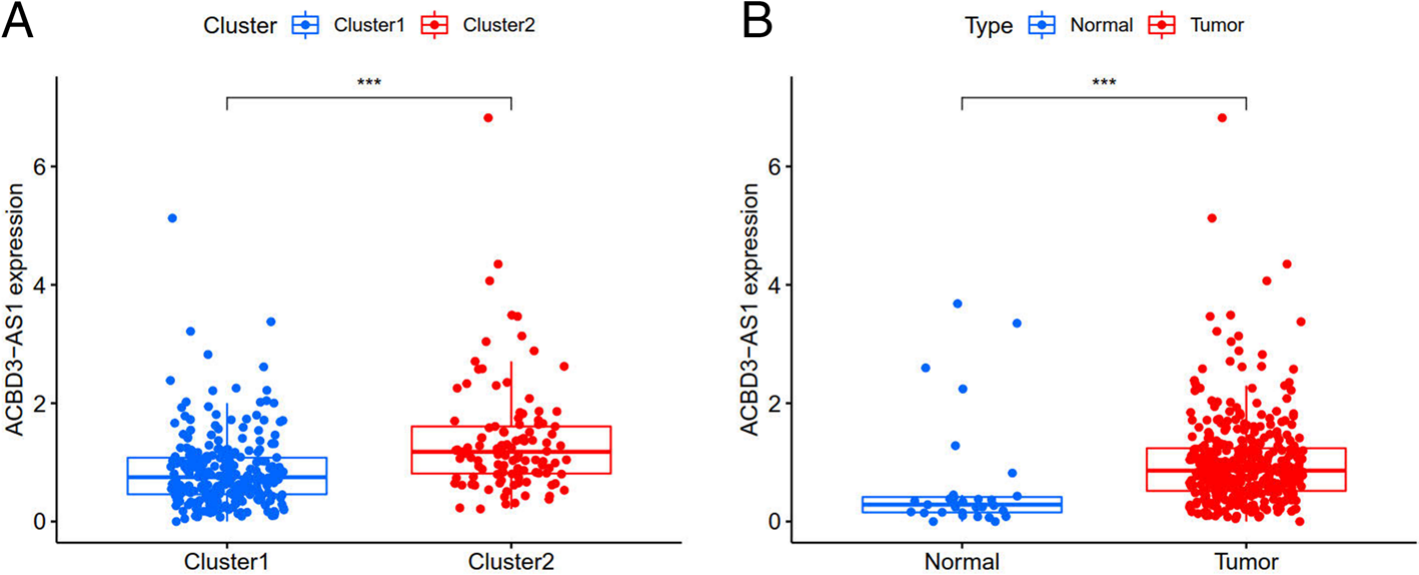
Difference expression of target gene in related subtype. The expression of ACBD3-AS1 in normal tissue is lower than tumor (**A**: *P* < 0.001); while the expression of above gene is higher in cluster 2 (**B**: *P* < 0.001)

**Fig. 6:**
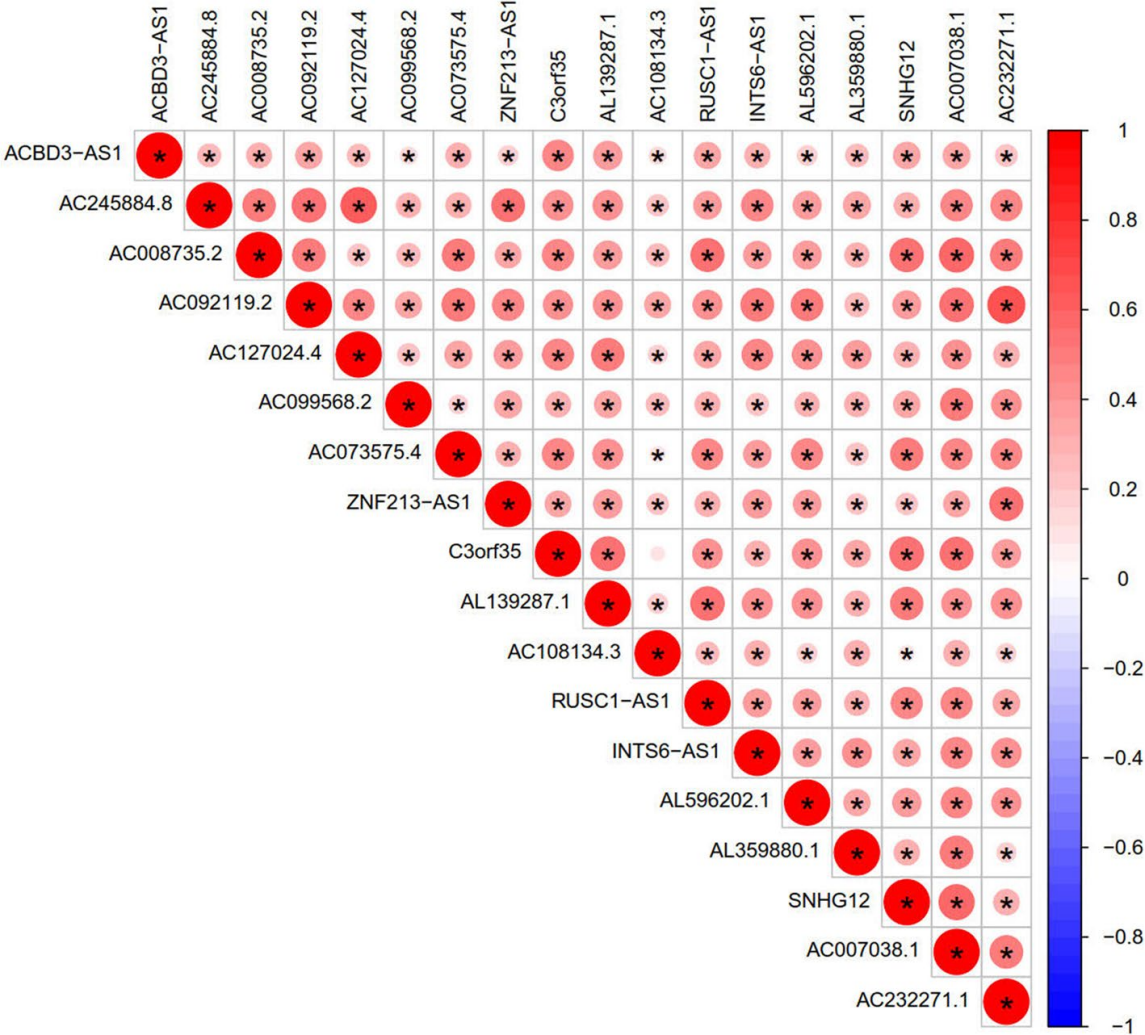
Correlation analysis of target gene and m6A lncRNAs. To analysis the correlation between target gene and prognostic m6A lncRNAs in gastric cancer. Red means positive correlation, while blue means negative correlation, * means the difference is statistically significant. (*P* < 0.05)

According to the expression of lncRNAs, when K = 2, there was least overlap between the two types, and the CDF value was lowest; therefore, we classified lncRNAs into two types: cluster 1 and cluster 2 in Fig. 3. Survival analysis according to the lncRNA subtypes was undertaken to evaluate the prognostic value of m6A-lncRNAs, and the survival rate of cluster 1 was higher than that of cluster 2 (*p* = 0.003), as shown in Fig. 4A. Heatmap depicts the differences that were identified in the expression of prognosis-related lncRNAs and the relationship was analyzed with regard to clinicopathological parameters (Fig. 4B). Some lncRNAs were highly expressed in cluster 1 whereas others were highly expressed in cluster 2. In Fig. 4B, there was no difference in the expression of prognosis-related lncRNAs in the different clusters. Differences in the expression of target genes in the related subtypes and in different types of tissue specimens are shown in Fig. 5. ACBD3-AS1 expression was lower in normal tissue than in the tumor (*p* < 0.001). Gene correlation analysis was conducted to ascertain the correlation between the target gene and the prognostic m6A-lncRNAs in GC (Fig. 6), and we found that the abovementioned target gene is related to prognosis-related m6A-lncRNA C3orf35 (*p* < 0.05).

### The Role of Immune Cell Infiltration and the Tumor Microenvironment

**Fig. 7:**
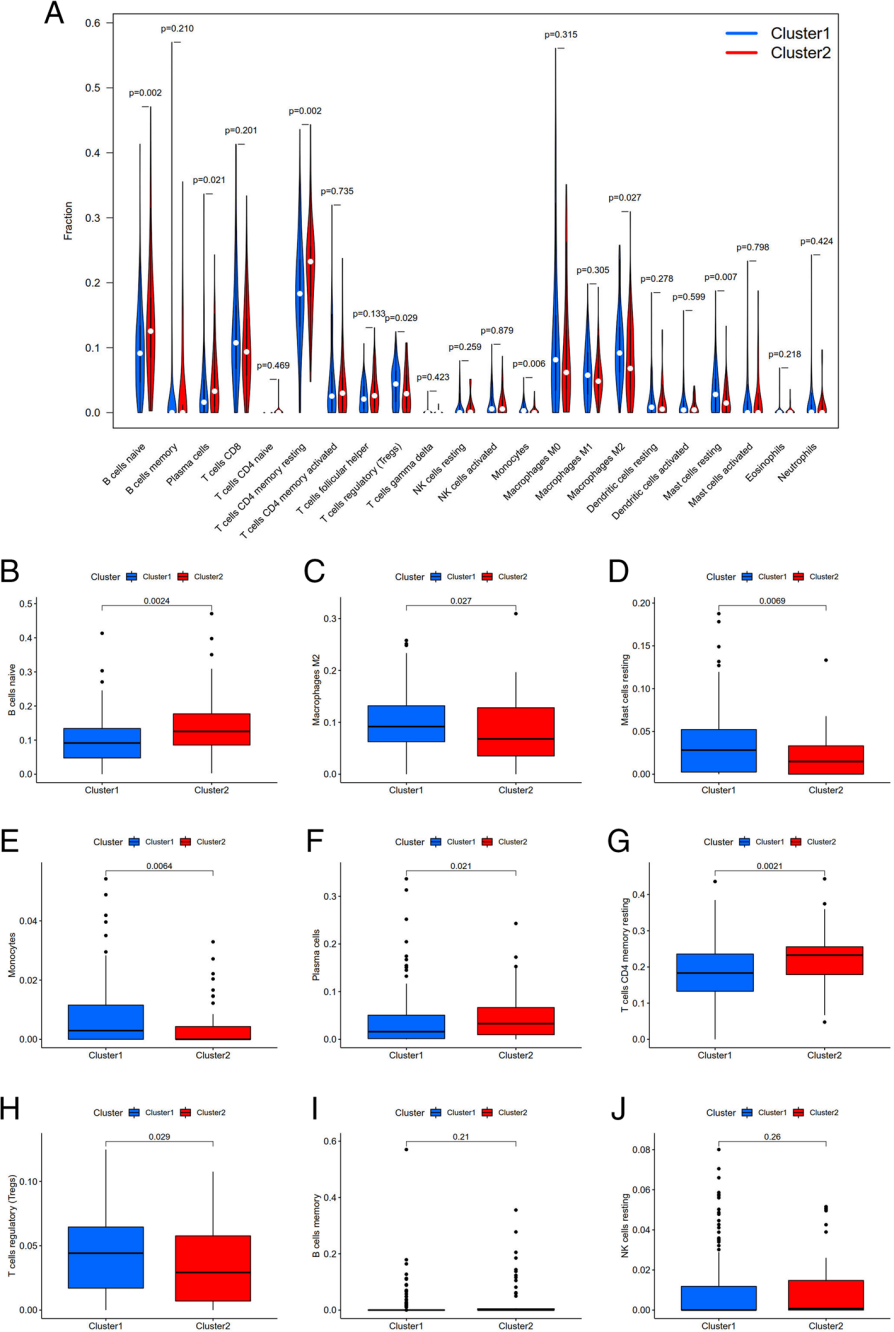
Difference analysis of immune cell infiltration in different cluster. **A**: Vioplot; **B**: Boxplot. Immune cells like Monocytes, Macrophages M2, Mast cells resting and T cell regulatory (Tregs) were higher in cluster 1; B cells naïve, Plasma cells, T cells CD4 memory resting were higher in cluster 2, *P* < 0.05

**Fig. 8:**
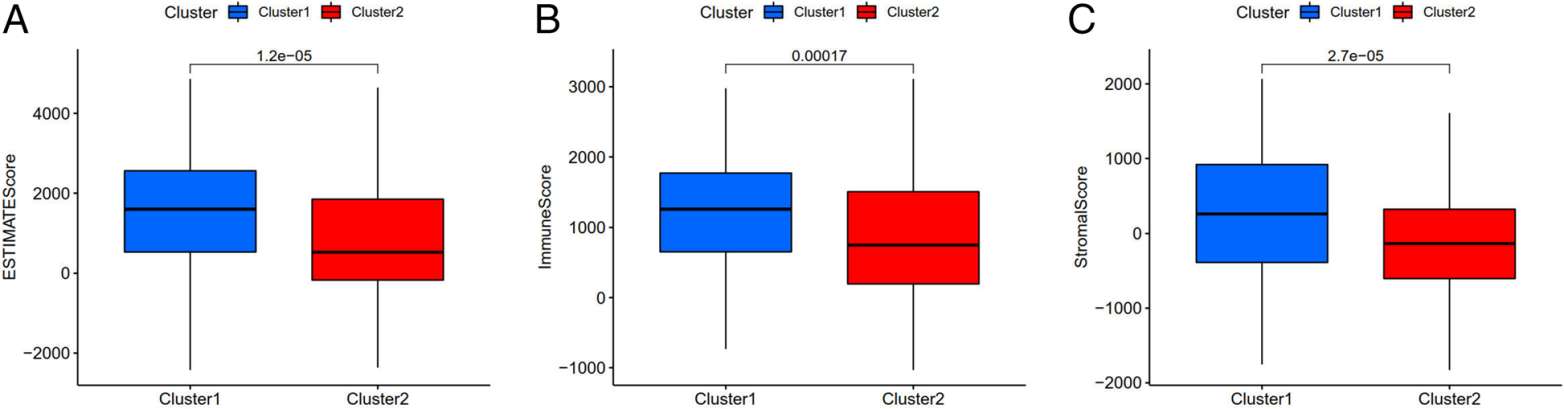
Difference analysis of tumor microenvironment in different subtype. All of the scores are higher in cluster 1, which means lower purity of tumor cell. (*P* < 0.05)

**Fig. 9:**
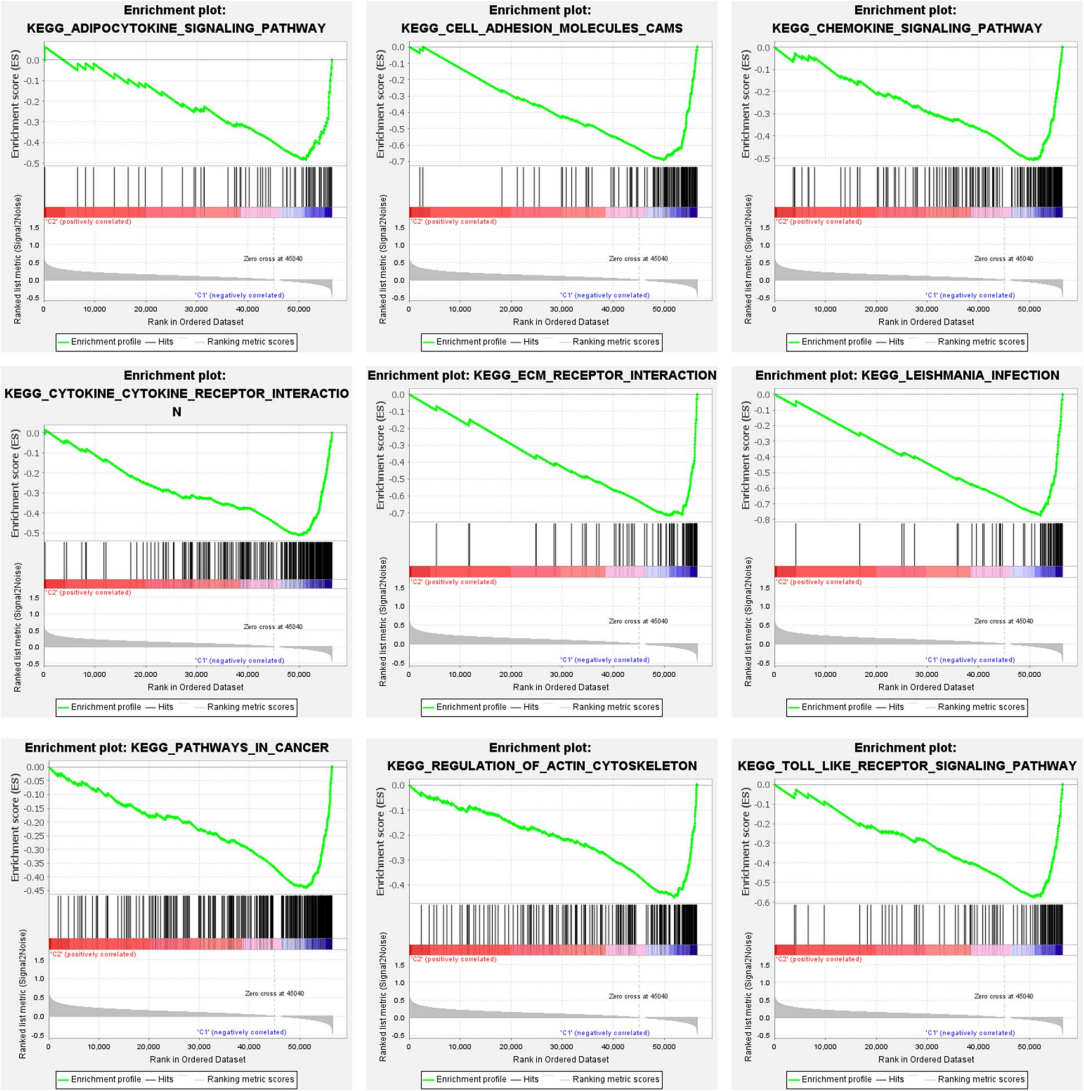
Gene set enrichment analysis. To clarify the difference of related function or pathway in different sample. Part of the results of GSEA are listed as above. Low risk of m6A related lncRNA were enriched in multiple cancer-related functions and pathways, which were upregulated in class C1. All of them are positive related to C1. Both FDR q-value and FWER *p*-Value < 0.05

To explore and calculate the infiltration of different immune cells in the samples, analysis of the tumor microenvironment was carried out. Differential analysis of immune cell infiltration in different clusters was carried out, and a vioplot was generated, as shown in Fig. 7A. Additionally, the infiltration level of each type of immune cell in each cluster of gastric cancer was analyzed, and a boxplot was drawn (Fig. 7B-J). As shown in the images, immune cells, such as B cell naïve, Plasma cells, resting memory CD4 T cell were highly clustered in cluster 2, while Macrophages M2, resting Mast cells, Monocytes, regulates T cells were highly clustered in cluster 1 (*P* < 0.05). We conducted a differential analysis of the tumor microenvironment in different subtypes of samples, and corresponding boxplots were generated to further explore the purity of tumor cells in the different types, as shown in Fig. 8. All of the scores were higher in cluster 1, indicating lower purity of the tumor cells and a higher level of immune-related cells in the tumor microenvironment (*P* < 0.05). Then, GSEA was conducted to clarify the differences in related functions and pathways in different samples, as shown in Fig. 9. The GSEA results are partially listed. Among the results, the most enriched gene set was “ADIPPOCYTOKINE SIGNALING PATHWAY”. All of the gene sets were positively associated with C1. Both the FDR q-value and the FWER *p*-values were < 0.05.

### m6a-related lncRNA Prognostic Model

**Fig. 10:**
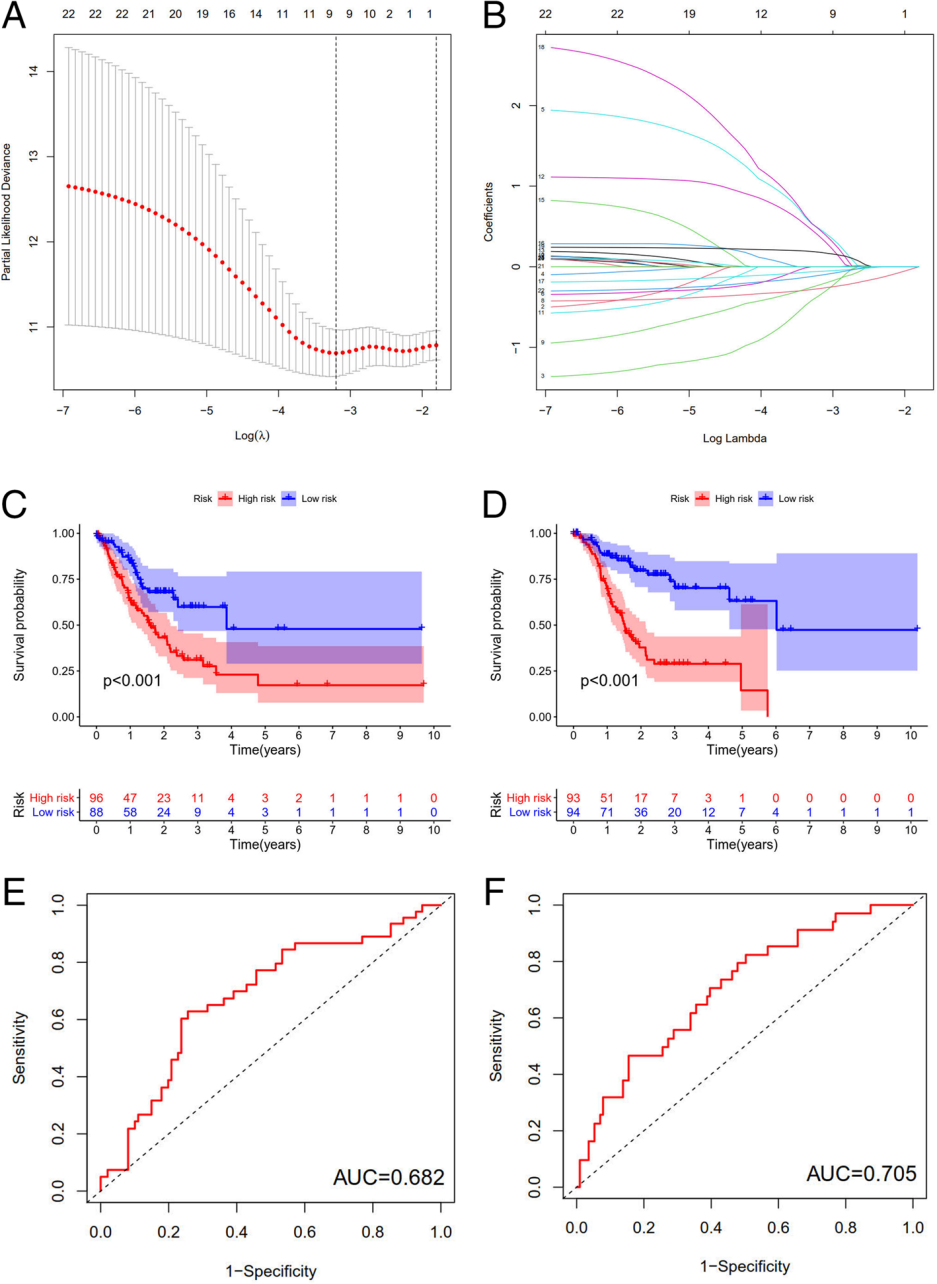
Prognostic model. **A** + **B**: Prognostic model was constructed via lasso regression. **C** + **D**: Survival curve of different groups. (C represents test group, while D represents train group, *P* < 0.001). **E** + **F**: ROC curve to evaluate the accuracy of our model to predict the survival of the disease. (The left represents test group, while right represents train group, AUC > 0.5)

**Fig. 11:**
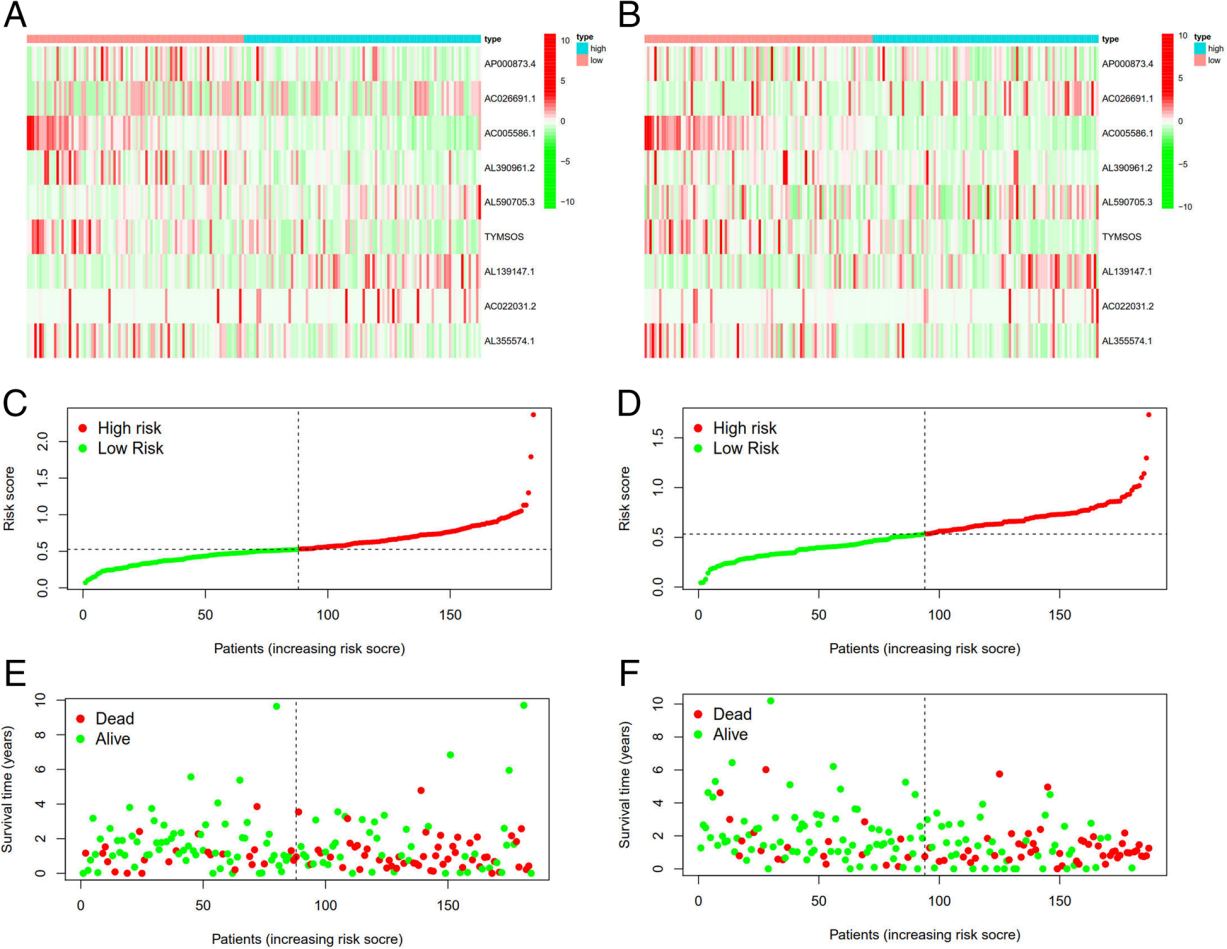
Risk related curve, heatmap and spot plot of test and train. With the risk score increases, the number of deaths increases,the ratio of high risk increases. **A** + **B** + **C**: test group, **D** + **E** + **F**: train group

**Fig. 12:**
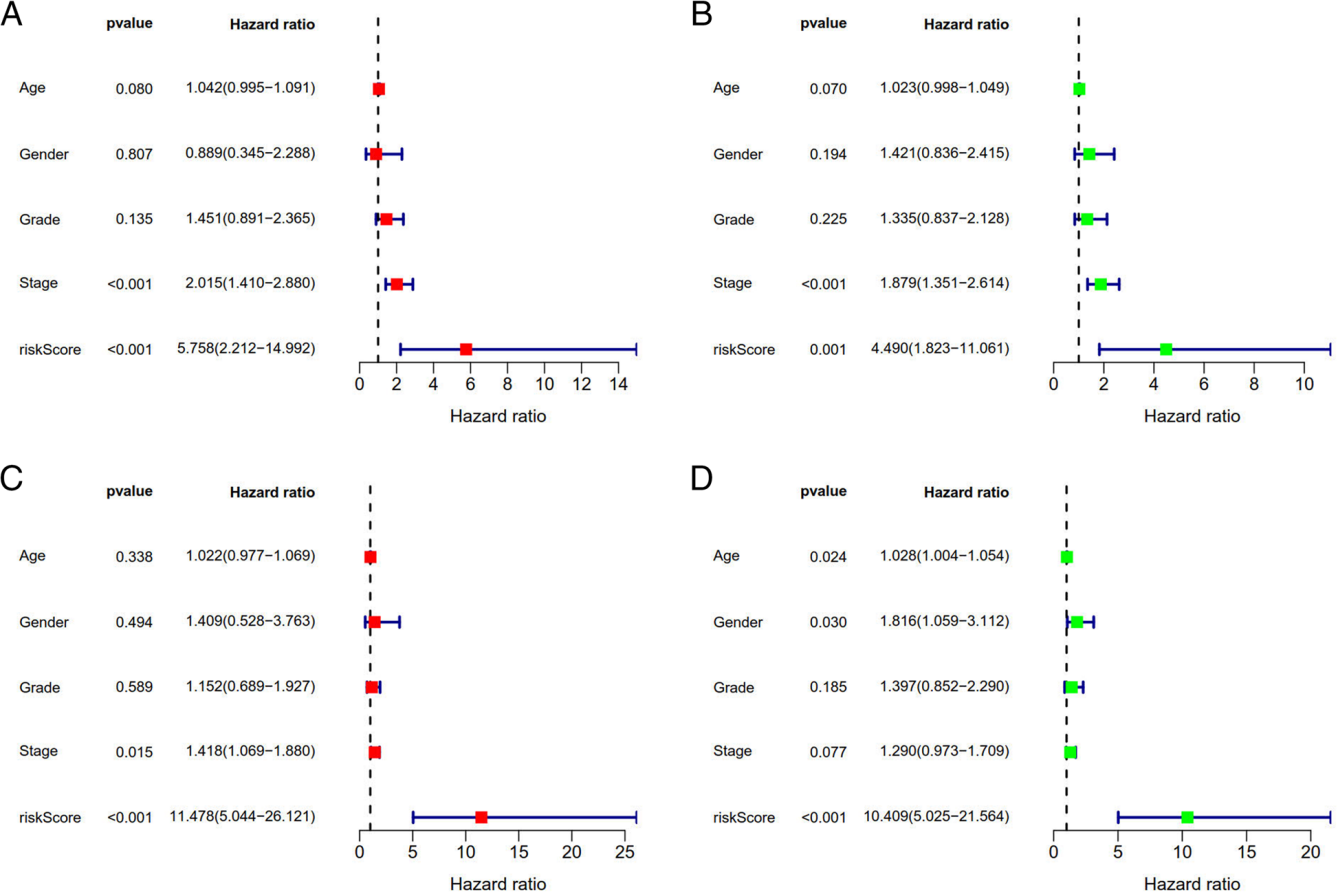
Multivariate and univariate analysis of independent prognostic analysis. (**A** + **B** represents test; **C** + **D** represents train, Stage ang risk Score were risk factors for the prognosis of GC; **A** + **C**: multivariate, **B** + **D**: univariate analysis), *P* < 0.05

**Fig. 13:**
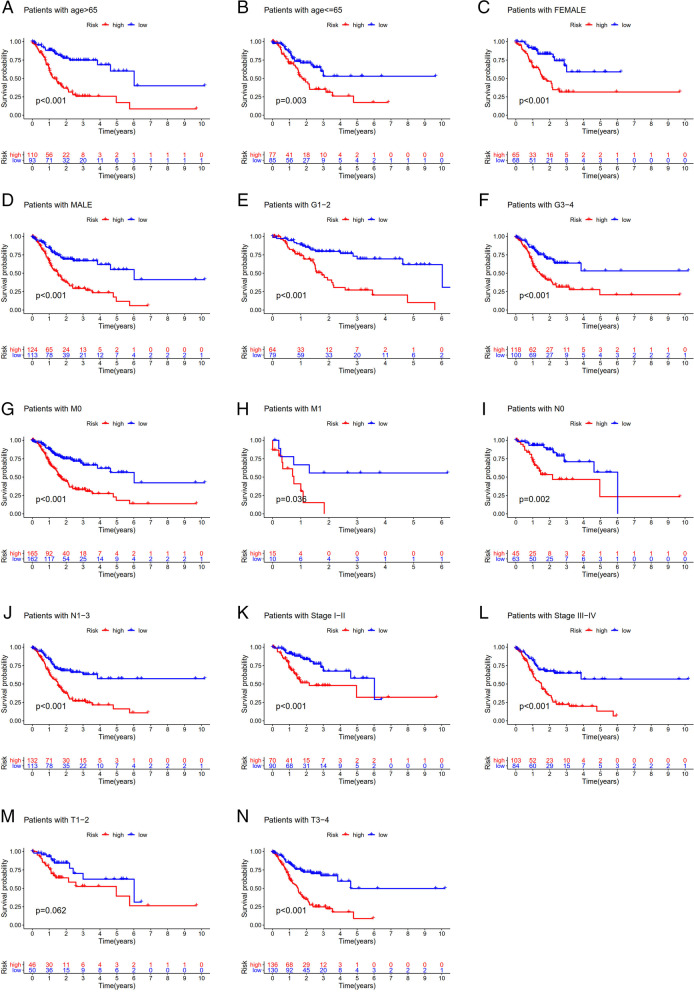
Survival curves for model validation. (our model could be applied to different clinical groups: age, gender, lymph node metastasis, stage and T stage, *P* < 0.05)

**Fig. 14:**
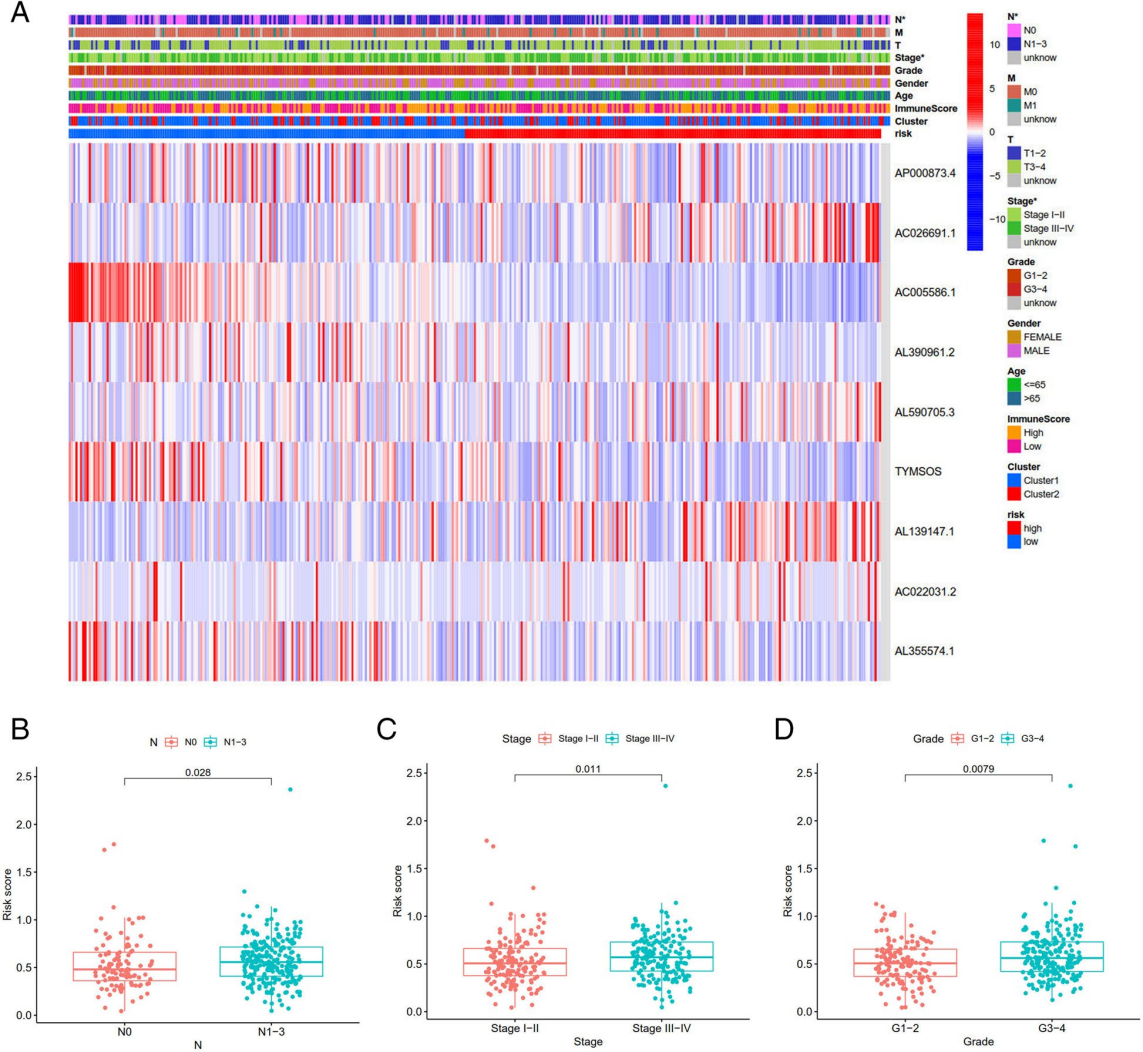
Risk and clinical correlation analysis. **A**: Heatmap of risk and clinical correlation analysis; **B**-**D**: Boxplot of risk and clinical correlation analysis. (Grade, N stage, Stage were closely related to risk score, *P* < 0.05)

**Fig. 15:**
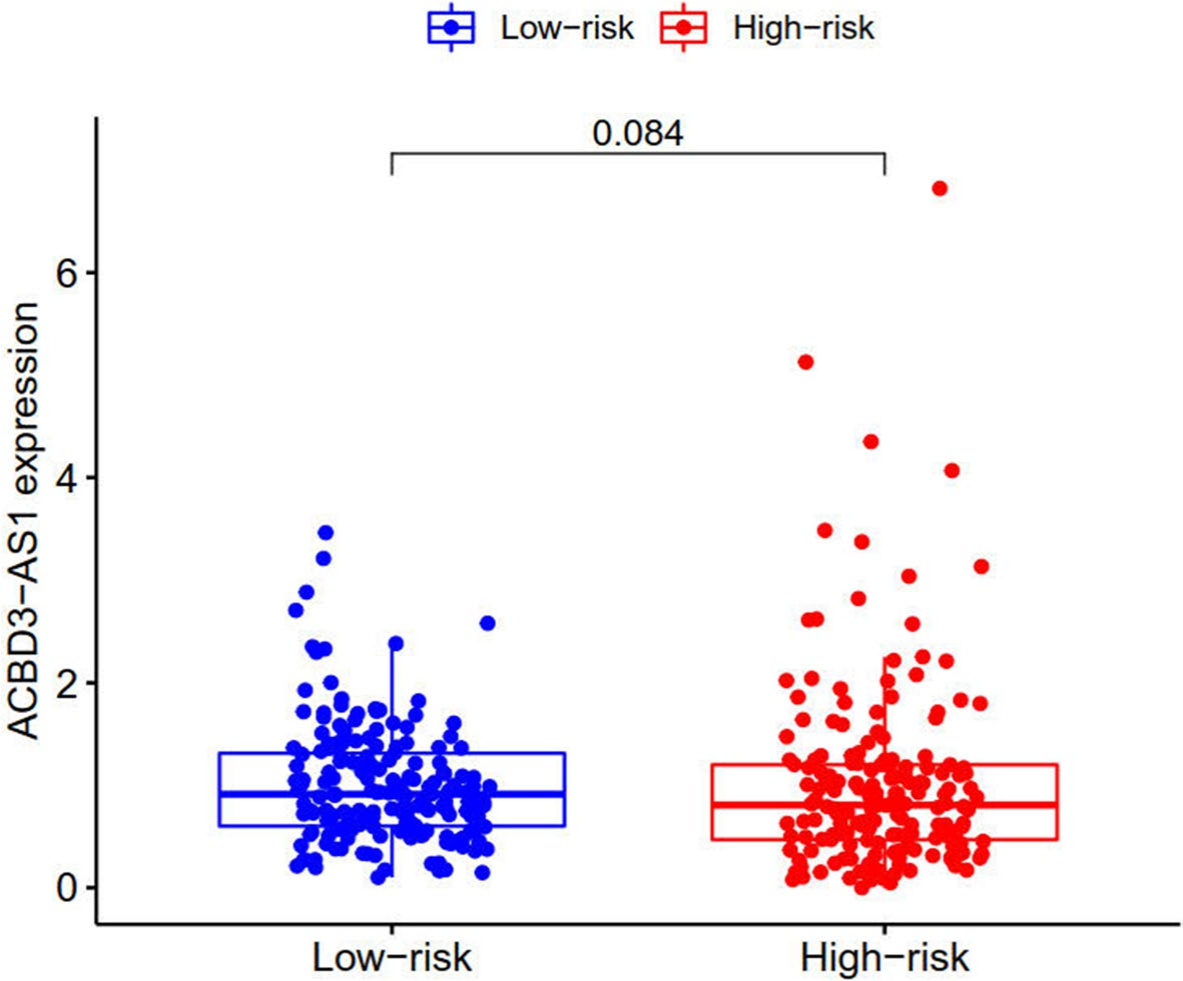
Genetic difference analysis of target gene. ACBD3-AS1 expression is no difference in different risk group. (*P* > 0.05)

**Fig. 16:**
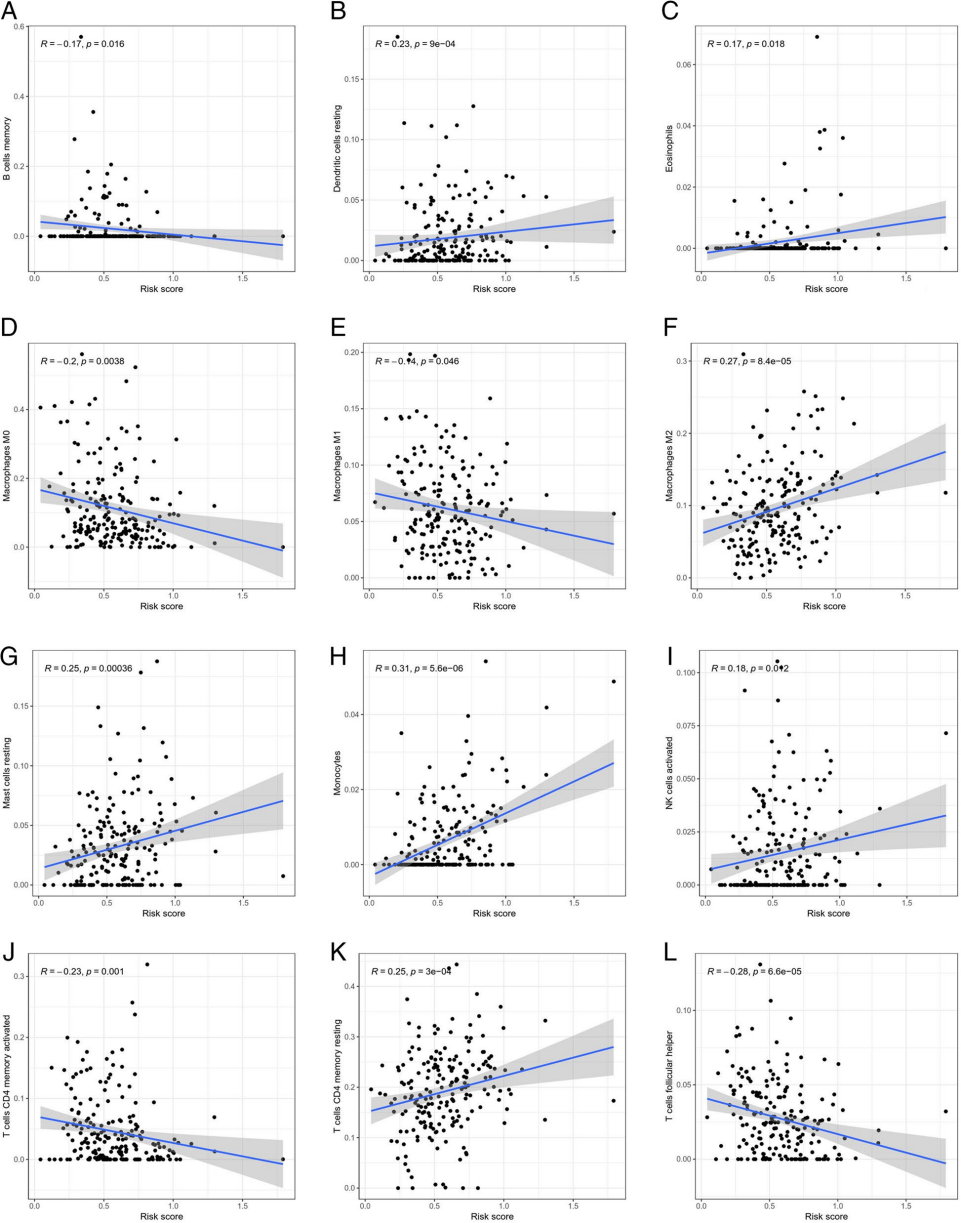
Scatter plots of correlation analysis of risk score and immune cells. Both T cells CD4 resting, NK cells activated, Monocytes, Mast cells resting, Macrophages M2, Eosinophils and Dendritic cells resting are positive related with risk score, R > 0 and *P* < 0.05.T cells follicular helper, T cells CD4 memory activated, Macrophages M1, Macrophages M0 and B cells memory are negative related with risk score, R < 0 and *P* < 0.05

Lasso regression was carried out to construct a prognostic model. All samples were divided into a high-risk group and a low-risk group according to the median value of the risk score of the prognostic m6A lncRNA. A training group (50%) and a test group (50%) were used for the lasso regression and related plots were generated, as shown in Fig. 10A + 10B. The survival curves of the different groups are depicted, and the high-risk and low-risk groups are compared in Fig. 10C + 10D. In both the test group and the training group, the survival rate of the low-risk subtype was higher than that of the high-risk subtype (P < 0.05). To evaluate the accuracy of our model for predicting the survival of patients with the disease, the corresponding ROC curve was generated via the time-ROC package (Fig. 10E + F). The ROC curve analysis revealed that both AUC values were > 0.5, indicating the considerable accuracy of our model for predicting the survival of patients with the disease. A risk curve was generated, and the survival status and risk of m6A lncRNAs were assessed based on the curve (Fig. 11). As the risk score increased, the number of deaths increased, and the ratio of high risk increased. The m6A prognosis-related lncRNA AC104819.3 was associated with low risk, while the other lncRNAs were associated with high risk. Independent prognostic analysis was conducted to evaluate whether our model was independent of other clinical prognostic factors that affect patient outcomes (Fig. 12). We found that age, stage and risk score were independent prognostic risk factors for gastric cancer (*P* < 0.05). Model validation for clinical groups was utilized to test and verify whether our model could be applied to different clinical parameters, as shown in Fig. 13. We found that our model could be applied to the following different clinical parameters: age, sex, lymph node metastasis, stage and T stage (*P* < 0.05). Risk and clinical correlation analysis was carried out to clarify related high-risk and low-risk m6A lncRNAs and expound the correlation of clinical characteristics and our prognostic risk model in Fig. 14A and B-D. Both AC026691.1, AL590705.3, AL139147.1 were highly expressed in high risk group, while AP000873.4, AC005586.1, AL390961.2, TYMSOS, AL355574.1 were lowly expressed in low risk group. Both Grade, N stage and Stage were closely related to risk score (*P* < 0.05). Genetic differential analysis was conducted to assess the expression difference of target genes in the different risk groups in our model in gastric cancer, as shown in Fig. 15. The expression of ACBD3-AS1 in no difference between different risk group (*P* = 0.084). A scatter plot was depicted to visualize this nexus and assess whether immune cells were beneficial or detrimental (Fig. 16). Both T cells CD4 resting, NK cells activated, Monocytes, Mast cells resting, Macrophages M2, Eosinophils and Dendritic cells resting were positively related to the risk score, R > 0 and P < 0.05.T cells follicular helper, T cells CD4 memory activated, Macrophages M1, Macrophages M0 and B cells memory are negatively related with risk score, R < 0 and P < 0.05.

## Discussion

The high incidence and mortality rate of GC seriously endangers human health. Surgery is considered to be the only radical cure for GC, but is prone to result in anastomotic leakage, intestinal obstruction, early recurrence and other serious complications, which seriously worsens the patient prognosis and reduces survival rates. m6A modification is accurately regulated by “writers”, “erasers”, and “readers”, which are involved in several mRNA metabolism pathways. Additionally, m6A modifications influence the processing of lncRNA. m6A modifications regulate cellular proliferation and maturation processes, both of which are linked with cancer development. Consequently, the regulation of m6A modification in cancer cells could have a profound impact on the progression of malignant tumor research [13]. An increasing number of studies have revealed the pathological significance of m6A in cancers [14, 15]. LncRNA is a major class of noncoding RNAs. LncRNA plays vital roles in chromatin organization and transcriptional and posttranscriptional regulation [16]. Previous studies have mainly focused on the correlation between specific m6A-related genes or pathways and the diagnosis and treatment of tumors; the lack of systematic analysis of m6A-related lncRNA in gastric cancer ought to be addressed. Therefore, recognition and analysis of m6A-related lncRNA in large cohorts of GC patients are of considerable significance, guiding latent directions and targets for GC research.

In the current study, we extracted m6A-related gene expression data and identified mRNAs and lncRNAs. Additionally, co-expression analysis was carried out to determine the correlation between m6A-related gene expression and lncRNAs. As shown in the co-expression network plot, we found an interesting phenomenon in which several lncRNAs were linked with m6A-related genes in GC. This finding fueled our interest in the expression of m6A-related lncRNAs and their related functions in GC. Prognosis-related lncRNAs were identified and the confidence interval and hazard ratio were calculated. Univariate Cox regression analysis indicated that m6A-related lncRNAs were closely related to the prognosis of GC. Cancers have many essential links with m6A modifications. m6A is considered to influence lncRNA splicing, which might alter cancer progression [17, 18]. There were 17 m6A prognosis-related lncRNAs in our study, the expression of which was different between tumor and normal tissues. Some lncRNAs were highly expressed in tumor tissues, while others were highly expressed in normal tissues (P < 0.05). It has been reported that HBXIP is upregulated in cancers, which plays a role as a tumor promoter in cancer via driving metabolic reprogramming through METTL3-mediated m6A modification [19]. Lately, it has been shown that HBXIP might promote the development of gastric cancer, which is m6A-modified [20]. It was recently identified that lncRNA RP11 expression is upregulated in gastric cancer and functions by promoting migration and invasion via epithelial–mesenchymal transition [16]. m6A modification promotes the upregulation of RP11 expression in GC cells by enhancing epigenetic etiology and pathogenesis of GC [21]. The modification of lncRNA may act an essential role in protein interactome and influence the progress of cancer [22]. The above findings might explain the result of our study in which we found that some m6A lncRNAs are overexpressed in tumors, while others are highly overexpressed in normal tissue. In addition, m6A lncRNAs may act as oncogenes or tumor suppressors.

We further explored the role of m6A lncRNA in GC. Survival analysis according to subtypes of lncRNAs was conducted to evaluate the prognostic value of m6A lncRNAs. Low-risk lncRNAs are beneficial to the prognosis of GC. In addition, lncRNAs are closely related to the survival rate of GC patients. The results of our study are consistent with the conclusion of Wang et al. that m6A-induced lncRNA RP11 expression triggers the malignancy and immunosuppression of gastric cancer cells via upregulation of YAP1 expression [16]. Additionally, Yang et al. [20] showed that the long noncoding RNA HBXIP promotes progression of gastric cancer through METTL3-mediated m6A modification of HIF-1α, also supporting that m6A lncRNA is closely related to the prognosis of GC. The expression of m6A lncRNAs in different clusters was not different, possibly because most of the m6A lncRNAs were expressed at low levels in GC in our study. In addition, studies on m6A modification of lncRNAs are still small in number. Thus, there is an urgent need for further research on lncRNA m6A modification and recognition to validate our results.

Gene ACBD3-AS1(ACBD3 antisense RNA 1) is a member of the lncRNAs located at Chromosome 1: 226,148,003–226,155,071 forward strand, which is ubiquitous expression in stomach, colon and 25 other tissues. Overexpression of ACBD3-AS1 increased feasibility and inhibited apoptosis via stimulating the expression of apoptosis related genes. Research illustrated that overexpression of ACBD3-AS1 launched accumulation of JAK2, indicating potential comic dialog between ACBD3-AS1 and the JAK2 signaling pathway, which revealed that this transcription factor could unleash tumor booster properties in gastric carcinoma [23]. In our study, the expression of ACBD3-AS1 is higher in GC tumor sample, indicating that it might be an oncogene. Additionally, the expression of ACBD3-AS1 was higher in cluster 2, which indicates it may be harmful to prognosis of gastric cancer. Its highly expression in various GC cell types might explain this result. As shown in the analysis of the correlation between the target genes and prognostic m6A lncRNAs in GC, ACBD3-AS1 is closely associated with several m6A lncRNAs. In addition, ACBD3-AS1 was most positively correlated with AC092119.2, AC007038.1, AL139287.1, SNHG12 and C3orf35. With the increased expression of the above m6A lncRNAs in GC cells, the expression of ACBD3-AS1 is increased. This phenomenon further supports our hypothesis that ACBD3-AS1 may be an oncogene. Additionally, the m6A lncRNAs might be therapeutic targets for GC. This is consistent with the result that under the condition of SNHG12 addition, gastric cancer cell proliferation, migration and invasion were notably heightened and cell apoptosis was lessened to accelerate the malignant progression of GC by activating the phosphatidylinositol 3-kinase/AKT pathway [24]. However, the specific molecular mechanism leading to tumorigenesis needs further research to clarify.

Moreover, we explored and calculated the infiltration of different immune cells in the samples to identify the role of immune cell infiltration and the tumor microenvironment in GC. Differential analysis of immune cell infiltration indicated that immune cells such as naïve B cell, Plasma cells, resting CD4 memory T cell were enriched in cluster 2, while Macrophages M2, resting Mast cells, Monocytes, regulates T cells were highly clustered in cluster 1. As stated before, cluster 2 represents high risk GC and poor prognoses. Therefore, the infiltration of naïve B cell, Plasma cells, resting CD4 memory T cell in the tumor microenvironment may be harmful to patient prognosis. Our conclusion is in line with the conclusion of Zhao et al*.,* who found that naïve B cell in cancer tissue correlated with tumor metastases and fully functional regulatory activity against human gastric cancer immunity [25]. Wu et al. suggested that resting CD4 memory T cell is unable to mount sufficient cytotoxic activity against GC cells, indicating that resting CD4 memory T cell is detrimental to GC patients [26]. Differential analysis of the tumor microenvironment in different subtypes was conducted to further explore the purity of tumor cells in the different clusters. All of the scores were higher in cluster 1, indicating lower tumor cell purity and more immune-related cells in the tumor microenvironment of cluster 1. This result is consistent with the conclusion that cluster 1 is low-risk and beneficial for patients. The results of Kemi encourage the use of immune cell score analysis to predict the prognosis of gastric cancer and high score improves the 5-survival rate of GC patients [27]. Research suggested that the immune microenvironments of metastatic tumors was less immunologically active compared to that of primary tumors in gastric cancer patients, which might help establish reliable prognostic signatures due to assessments of stromal and immune components [28]. The results of both studies support our hypothesis that immune cell infiltration in the tumor microenvironment influences the prognosis of GC patients. The higher the immune score is, the lower the purity of the tumor and eventually the better the prognosis.

Next, we carried out GSEA. “ADIPPOCYTOKINE SIGNALING PATHWAY” was the most significantly enriched gene set. Regrettably, there are little researches about this signaling pathway in gastric cancer. As a result, it may be our next research direction to further explore the diagnosis and therapy of GC. Taking into account the above factors, m6A lncRNA may play its function by regulating the “ADIPPOCYTOKINE SIGNALING PATHWAY” to influence the migration and proliferation of GC cells. An m6A lncRNA-related prognostic model was constructed via lasso regression. In both the test group and the training group, the survival rate of the low-risk subtype was higher than that of the high-risk subtype. An m6A lncRNA-related prognostic model can therefore predict the outcome of GC. Additionally, the accuracy of our model for predicting the survival of patients with the disease is considerable. As the risk score increased, the number of deaths increased, and the ratio of high risk increased. Furthermore, our model was independent of other clinical prognostic factors that affect patient outcomes. Also, the model could be applied to different clinical groups. m6A modification of lncRNAs may change the structure of lncRNAs and affect their interaction with proteins, which may mediate gene transcription repression [29, 30]. m6A modification of lncRNAs possibly alters their subcellular dissemination*,* which regulates lncRNA stability and promotes tumorigenesis and metastasis [30]. In summary, the literature and our research results identified that m6A lncRNA could be a suitable clinical model to predict the outcome of GC. The results of genetic differential analysis indicated that the expression of the ACBD3-AS1 gene was higher in the high-risk group in our model in gastric cancer, which further confirmed that ACBD3-AS1 may be an oncogene in GC. As there are few studies on the ACBD3-AS1 gene and no related study has explored the role of ACBD3-AS1 in GC cells, the results need further study for clarification. Correlation analysis of risk and immune cells was conducted to evaluate the relationship among immune cells and the risk score. Macrophages M2 was positively correlated with the risk score. The conclusion of Takahisa clarified our result that M2 phenotype could contribute to tumor progression and is expected to be a promising target in the treatment of gastric cancer [31]. This finding is consistent with the results of the differential analysis of immune cell infiltration in the different clusters. Resting mast cells might disturb the anti-tumor immune response, and the function might differ depending on its composition via working together with BicC family RNA-binding protein 1 [32].

## Conclusion

Overall, we confirmed the prognostic value of m6A lncRNA by analyzing the expression profiles and clinical data of gastric cancer samples from the TCGA database. An immune cell infiltration and prognosis-related model was conducted to confirm the role of m6A-related lncRNA in gastric cancer. Functional and pathway GSEA further identified involved pathways. Hereafter, determined that ACBD3-AS1 is highly related to the overall survival rate of GC patients. Differential expression analysis and correlation analysis further clarified the prognostic value of ACBD3-AS1. ACBD3-AS1 is most positively correlated with AC092119.2, AC007038.1, AL139287.1, SNHG12 and C3orf35. Both resting CD4 T cells, activated NK cells, Monocytes, resting Mast cells, Macrophages M2, Eosinophils and resting Dendritic cells cause deterioration of prognosis. Follicular helper T cells, activated CD4 memory T cells, Macrophages M1, Macrophages M0 and memory B cells are positively related to the prognosis of GC. Our study reinforces the understanding of m6A-related lncRNA and provides novel therapeutic targets and prognosis-related biomarkers for further research.

## Data Availability

The dataset supporting the conclusions of this article is available upon reasonable request at the TCGA.

## Abbreviations

GC: Gastric cancer
lncRNA: Long non-coding RNA
m6A: N6-methyladenosine
TME: Tumor microenvironment
TCGA: The Cancer Genome Atlas
KEGG: Kyoto Encyclopedia of Genes and Genomes
GSEA: Gene sets enrichment analysis
ACBD3-AS1: ACBD3 antisense RNA 1
